# Dexmedetomidine Reduces Isoflurane-Induced Neuroapoptosis Partly by Preserving PI3K/Akt Pathway in the Hippocampus of Neonatal Rats

**DOI:** 10.1371/journal.pone.0093639

**Published:** 2014-04-17

**Authors:** Yujuan Li, Minting Zeng, Weiqiang Chen, Chuiliang Liu, Fei Wang, Xue Han, Zhiyi Zuo, Shuling Peng

**Affiliations:** 1 Department of Anesthesiology, Sun Yat-sen Memorial Hospital, Sun Yat-sen University, Guangzhou, Guangdong, China; 2 Department of Anesthesiology, Affiliated Shantou Hospital of Sun Yat-sen University, Shantou, Guangdong, China; 3 Department of Anesthesiology, ChanCheng Center Hospital, Foshan, Guangdong, China; 4 Department of Anesthesiology, University of Virginia Health System, Charlottesville, Virginia, United States of America; Massachusetts General Hospital, United States of America

## Abstract

Prolonged exposure to volatile anesthetics, such as isoflurane and sevoflurane, causes neurodegeneration in the developing animal brains. Recent studies showed that dexmedetomidine, a selective α2-adrenergic agonist, reduced isoflurane-induced cognitive impairment and neuroapoptosis. However, the mechanisms for the effect are not completely clear. Thus, we investigated whether exposure to isoflurane or sevoflurane at an equivalent dose for anesthesia during brain development causes different degrees of neuroapoptosis and whether this neuroapoptosis is reduced by dexmedetomidine via effects on PI3K/Akt pathway that can regulate cell survival. Seven-day-old (P7) neonatal Sprague-Dawley rats were randomly exposed to 0.75% isoflurane, 1.2% sevoflurane or air for 6 h. Activated caspase-3 was detected by immunohistochemistry and Western blotting. Phospho-Akt, phospho-Bad, Akt, Bad and Bcl-xL proteins were detected by Western blotting in the hippocampus at the end of exposure. Also, P7 rats were pretreated with various concentrations of dexmedetomidine alone or together with PI3K inhibitor LY294002, and then exposed to 0.75% isoflurane. Terminal deoxyribonucleotide transferase-mediated dUTP nick end labeling (TUNEL) and activated caspase-3 were used to detect neuronal apoptosis in their hippocampus. Isoflurane, not sevoflurane at the equivalent dose, induced significant neuroapoptosis, decreased the levels of phospho-Akt and phospho-Bad proteins, increased the expression of Bad protein and reduced the ratio of Bcl-xL/Bad in the hippocampus. Dexmedetomidine pretreatment dose-dependently inhibited isoflurane-induced neuroapoptosis and restored protein expression of phospho-Akt and Bad as well as the Bcl-xL/Bad ratio induced by isoflurane. Pretreatment with single dose of 75 µg/kg dexmedetomidine provided a protective effect similar to that with three doses of 25 µg/kg dexmedetomidine. Moreover, LY294002, partly inhibited neuroprotection of dexmedetomidine. Our results suggest that dexmedetomidine pretreatment provides neuroprotection against isoflurane-induced neuroapoptosis in the hippocampus of neonatal rats by preserving PI3K/Akt pathway activity.

## Introduction

Volatile anesthetics, such as isoflurane and sevoflurane, are used in millions of young children every year during surgical procedures and imaging studies [1]. Recent studies have demonstrated that prolonged exposure to isoflurane and sevoflurane causes neurodegeneration in the developing animal brains and persistent learning deficits [2], [3], [4], [5], [6], [7], [8]. Isoflurane at an equivalent dose for anesthesia induces more profound apoptosis than sevoflurane in neonatal rat brains [9]. Some clinical retrospective studies have indicated that anesthesia and surgery in children younger than 4 years probably increase their probability of developing disabilities in reading, writing and math learning [10], [11]. These findings have led to the concern about the possible detrimental effects of anesthesia and sedation in the pediatric population. Therefore, it is important to explore the mechanisms of anesthesia-induced neurodegeneration and to develop potential protective strategies for it.

Dexmedetomidine (Dex), an α2-adrenergic agonist, has been developed for human clinical use as an anesthetic and sedative. Several studies have shown that dexmedetomidine has neuroprotective effects against ischemic cerebral injury through its activation of α2-adrenergic receptors and its binding at imidazoline 1 and 2 receptors [12], [13]. Besides, dexmedetomidine can increase the antiapoptotic proteins B cell lymphoma/leukemia-2 (Bcl-2) and murine double minute-2 (Mdm-2) and reduce the proapoptotic proteins Bax and p53 in a model of adult ischemic cerebral injury [14]. Dexmedetomidine also increases the phosphorylation of focal adhesion kinase (FAK) in the hippocampal slices subjected to oxygen-glucose deprivation (OGD) [15] and increases the basal levels of phosphorylated extracellular signal-regulated kinase 1/2 (ERK1/2) by means of an α2-adrenoceptor-independent mechanism [13]. Recently, Sanders and co-authors report that dexmedetomidine attenuates isoflurane-induced neurocognitive impairment and neuroapoptosis in neonatal rats partly by activating α2-adrenergic receptor [16], [17]. Whether there were other mechanisms underlying dexmedetomidine-caused neuroprotection against anesthetic-induced impairment in the developing rat brains is still not clear.

The Bcl-2 family is composed of proapoptotic proteins and antiapoptotic proteins. The balance of these proteins regulates mitochondrial membrane integrity and the release of apoptogenic factors from mitochondria, which has a critical impact on cell survival and death [18]. Bcl-associated death protein (Bad) is a pro-apoptotic protein that is activated through phosphorylation on its serine residues [19]. Phosphorylation of Bad by Proto-oncogene proteins c-akt (Akt) leads to the binding of Bad with the cytosolic protein 14-3-3 to release anti-apoptotic protein Bcl-xL, which blocks apoptosis by binding to the pro-apoptotic protein Bax [20], [21], [22]. Bcl-2 and Bcl-xL block Bax translocation to the mitochondria, maintain mitochondrial membrane potential, and prevent the release of cytochrome c from the mitochondria and the subsequent apoptosis [22].

Previous study has demonstrated isoflurane, not sevoflurane, induces neurotoxicity by decreasing the ratio of Bcl-2/Bax in the pheochromocytoma 12 (PC12) cells and primary cortical neurons [23]. Whether isoflurane and sevoflurane have a different effect on disturbing the expression of Bcl-xL and Bad as well as the Akt activation is undetermined. We hypothesize that isoflurane at equivalent dose for anesthesia induces more profound apoptosis than sevoflurane in the neonatal rat brains by disturbing the PI3K/Akt pathway and decreasing the ratio of Bcl-xL/Bad and that dexmedetomidine reverses these isoflurane-induced protein changes to provide its neuroprotection.

## Materials and Methods

This study was approved by the animal care committee at Sun Yat-sen University and performed in strict accordance with the National Institutes of Health Guide for the Use of Laboratory Animals. All efforts were made to minimize suffering.

Seven-day-old (P7) Sprague-Dawley rat pups (Guangdong Medical Laboratory Animal Co, China, permission number: SCXK 2008-0002) weighting 14–18 g were used because this is the time when they are very vulnerable to anesthesia-induced neuronal damage [2]. Rats were exposed for 6 h to 0.75% isoflurane or 1.2% sevoflurane (approximately 0.3 MAC in P7 rats as determined by Orliaguet et al [24]) in 30% oxygen or air in a temperature-controlled chamber as we described before [7], [25].

### Experimental protocol

Three experiments were performed. In experiment one, P7 rats were randomly assigned to expose to isoflurane, sevoflurane or air for 6 h. At the end of the exposure, some rats were used for blood sampling from the left cardiac ventricle for blood gas and glucose analysis (*n* = 8). Some rats were sacrificed immediately and their hippocampi were used for Western blot studies (*n* = 5). The rest rats were used for activated caspase-3 immunohistochemistry at 2 h after the termination of anesthesia (*n* = 10).

In experiment two, 25, 50 or 75 µg/kg dexmedetomidine or saline in 150 µl were administered by intraperitoneal injection 20 min before the exposure to isoflurane. One group received 25 µg/kg dexmedetomidine in 3 doses during the 6-h isoflurane exposure (at 0, 2, and 4 h after the onset of isoflurane expsoure), which was used as the positive control of neuroprotection of dexmedetomidine. Additionally, 75 µg/kg dexmedetomidine and three doses of 25 µg/kg dexmedetomidine were given alone to determine whether high doses of dexmedetomidine could induce apoptosis. Rats after these various treatment were used for TUNEL or activated caspase-3 immunohistochemistry staining (*n* = 12) as well as Western blot study (*n* = 7) as we described in experimental one.

In experiment three, rats received intracerebroventricular (i.c.v.) LY294002 [26] (Selleck Chemicals LLC, Houston, TX, USA) 20 min before pretreatment with 75 µg/kg dexmedetomidine and then isoflurane exposure. The intracerebroventricular injection was performed as we and others described before under isoflurane anesthesia [25], [27]. Vehicle (5 µl 10% DMSO) or LY294002 (25 µg/5 µl) was injected into the left lateral ventricle (stereotaxic coordinates: 2.0 mm rostral and 1.5 mm lateral to the lambda and 2.0 mm deep to the skull surface) using a 5 µl Hamilton syringe at a constant rate of 2.5 µl/min. The position of i.c.v. injection was verified by methylene blue in our preliminary experiments. Rats were sacrificed at the end of gas exposure. Their hippocampi were used for Western blot study (*n* = 8).

### Tissue Preparation

For Western blot studies, rat pups were sacrificed by decapitation at the end of the exposure. Hippocampi were isolated immediately on ice and then stored at −80°C until used. For immunohistochemistry and TUNEL studies, rat pups were anesthetized with isoflurane and perfused transcardially with ice-cold saline followed by 4% paraformaldehyde in 0.1 M phosphate buffer. Their brains were post-fixed for 48 h at 4°C and paraffin embedded and sectioned at 8 µm thickness. As we described before [7], [25], [28] at least three sections (100 µm apart among them) corresponding to Figures 95–97 in the Atlas of the Developing Rat Brain [29] for each animal were chosen for detecting cells with positive staining of activated caspase-3 or TUNEL.

### Immunohistochemistry Staining

Immunohistochemistry was performed as we have described previously [7], [28]. In brief, sections were incubated at 4°C overnight with an anti-cleaved caspase-3 primary antibody (1∶200; monoclonal antibody, Cell Signaling Technology, Beverly, MA, USA), followed by incubation with a secondary antibody (1∶200, Santa Cruz Biotechnology, Inc., Santa Cruz, CA, USA) for 40 min and the avidine-biotinylated peroxidase complex (Vectostain ABC-Kit, Vector Lab, Burlingame, CA, USA) for 40 min. Tissue sections were colorized with diaminobenzidine (DAB, Vector Laboratories, Burlingame, CA, USA). Cleaved caspase-3 positive cells in the hippocampal CA1, CA3 and dentate gyrus (DG) were analyzed with NIS-Elements BR imaging processing and analysis software (Nikon Corporation, Japan). The density of cleaved caspase-3 positive cells in the three hippocampal regions was calculated by dividing the number of caspase-3 positive cells by the area of that brain region.

### TUNEL Fluorescent Assay

TUNEL fluorescent assay was performed using the Dead End TM fluorometric TUNEL system kit (Promega, Madison, WI, USA) as we have described previously [25]. In brief, the slides were protected from direct light during experiment and Hoechst was used to stain nuclei. TUNEL positive cells in the hippocampal CA1 region were analyzed as mentioned in the immunohistochemistry staining. The density of TUNEL positive cells in CA1 region was calculated by dividing the number of TUNEL positive cells by the area of that brain region.

### Immunoblotting

Western blotting was performed as we have described previously [7], [25]. In brief, protein concentrations of samples were determined using the BCA protein assay (Bio-Rad, Hemel Hempstead, Herts, UK). Sixty micrograms of each protein sample were subjected to Western blot analysis using the following primary antibodies: anti-cleaved caspase-3 at 1∶2000 dilution, anti-phospho-Akt at 1∶1000 dilution, anti-Akt at 1∶2000 dilution, anti-phospho-Bad at 1∶1000 dilution, anti-Bad at 1∶1000 dilution, anti-Bcl-xl at 1∶2000 dilution and anti-β-actin at 1∶2000 dilution. All antibodies were purchased from Cell Signaling Technology, Beverly, MA, USA. Images were scanned by an Image Master II scanner (GE Healthcare, Milwaukee, WI, USA) and were analyzed using ImageQuant TL software v2003.03(GE Healthcare, Milwaukee, WI, USA). The protein expression of phospho-Akt or phospho-Bad was normalized to their total Akt or Bad, respectively. The band signals of other interesting proteins were normalized to those of the corresponding β-actin and then expressed as fractions of control sample from the same gels.

### Statistical Analysis

Sample size was calculated to detect a difference of 40% between means with an 80% power at a significance level of 0.05 using the PASS 11 software. We chose to detect a 40% difference in means because 40% was the least difference in means in our preliminary studies on phospho-Akt protein expression.

All residuals followed normal distribution as detected by Shapiro–Wilk test. Data of caspase-3 immunohistochemical staining and TUNEL positive cells had significant heterogeneity of variance as detected by Levene's test. They were expressed as median (95% confidence interval) and were compared using the nonparametric Kruskal–Wallis test followed by Dunn's multiple comparison test [30]. Data of Western blots for caspase-3, Akt, Bad and Bcl-xl as well as blood gas and glucose levels had no significant heterogeneity of variance. These data were expressed as mean ± SD and analyzed by one-way ANOVA, and all possible comparisons between groups were made using Student's two-group t-tests with all P-values Bonferronni adjusted. The GraphPad Prism 6.0 software was used to conduct the statistical analysis. All P-values were corrected. Statistical significance was accepted at *P*<0.05.

## Results

In experiment one, the blood gas value of rats did not change significantly during the 6-h exposure to 0.75% isoflurane or 1.2% sevoflurane, suggesting that anesthetic gas exposure at this dose did not inhibit ventilation/oxygenation of neonatal rat (Table 1). There was no hypoglycemia in the three groups of rats. Isoflurane at high concentration (2.3%) can induce hypoglycemia, which may contribute to the neurodegeneration of neonatal rodents [31]. Our results found that isoflurane at low concentration (0.75%) did not decrease the level of blood glucose significantly (Table 1), which indicates that the neuroapoptosis and protein change induced by isoflurane as described below are not related to hypoglycemia. No rats died during or after gas exposure in all experiments.

**Table 1:** Arterial Blood Gases and Glucose levels.

	pH	PO_2_ (mmHg)	PCO_2_ (mmHg)	Blood glucose (mmol/L)
**Control**	7.45±0.06	101±5	40±2	5.39±0.67
**Isoflurane**	7.37±0.13	108±7	42±4	8.62±0.88* #
**Sevoflurane**	7.38±0.08	105±6	42±3	5.42±1.33

Values are mean ± S.D.

*P<0.001 versus Control;

# *P*<0.001 versus Sevoflurane by one-way ANOVA and post-hoc Bonferronni' correction, n = 8.

### Isoflurane, Not Sevoflurane, Induced Neuroapoptosis and Inhibited Akt Activity in Hippocampus

**Figure 1:**
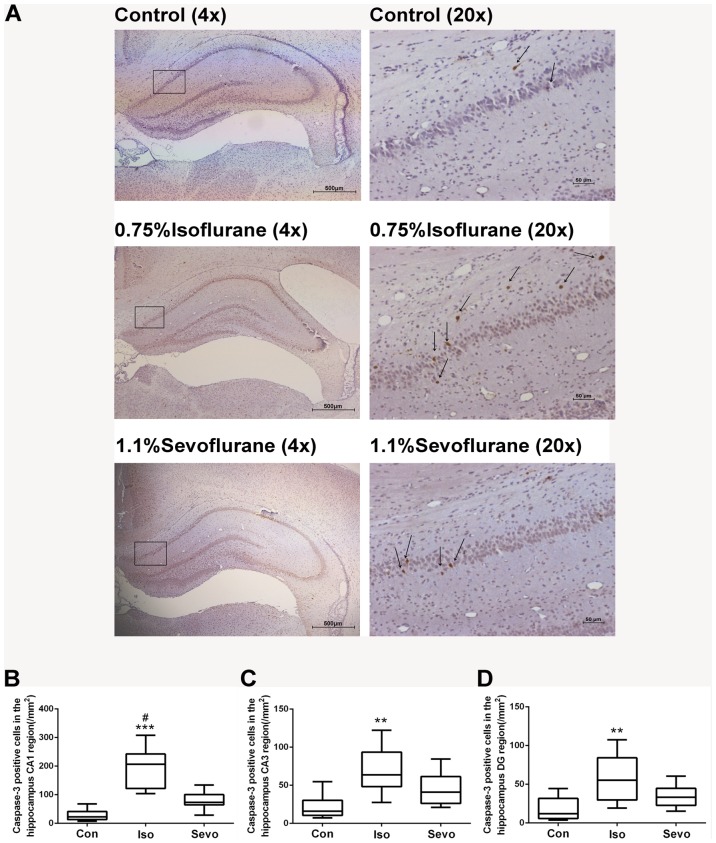
Isoflurane, not sevoflurane, induced neuroapoptosis in the hippocampus of P7 rats (n = 10 in each group). (A) Representative images of cleaved caspase-3 immunohistochemical (IHC) staining in the hippocampal CA1 region from various experimental groups and the sample sections were illustrated at different magnifications. Arrows indicate caspase-3 positive cells. Representative CA1 region with high magnification located within the rectangles illustrated on the left panel demonstrating whole hippocampus of neonatal mice developing brains at low magnification. Scan bar = 500 µm for the low magnification on the left panel and 50 µm for the high magnification on the right panel of brain sections. (B,C,D) Box plots represent quantitative analysis of cleaved caspase-3 positive cells in the hippocampal CA1 (B), CA3 (C) and DG (D) regions by the Kruskal–Wallis test with the Dunn test for post hoc comparisons. Horizontal lines: group medians; boxes: 25th–75th percentile; whiskers: 5th–95th percentile. Con: control, Iso: isoflurane, Sevo: sevoflurane. **P<0.01, ***P<0.0001 versus control group; ^#^ *P*<0.05 versus sevoflurane group.

**Figure 2:**
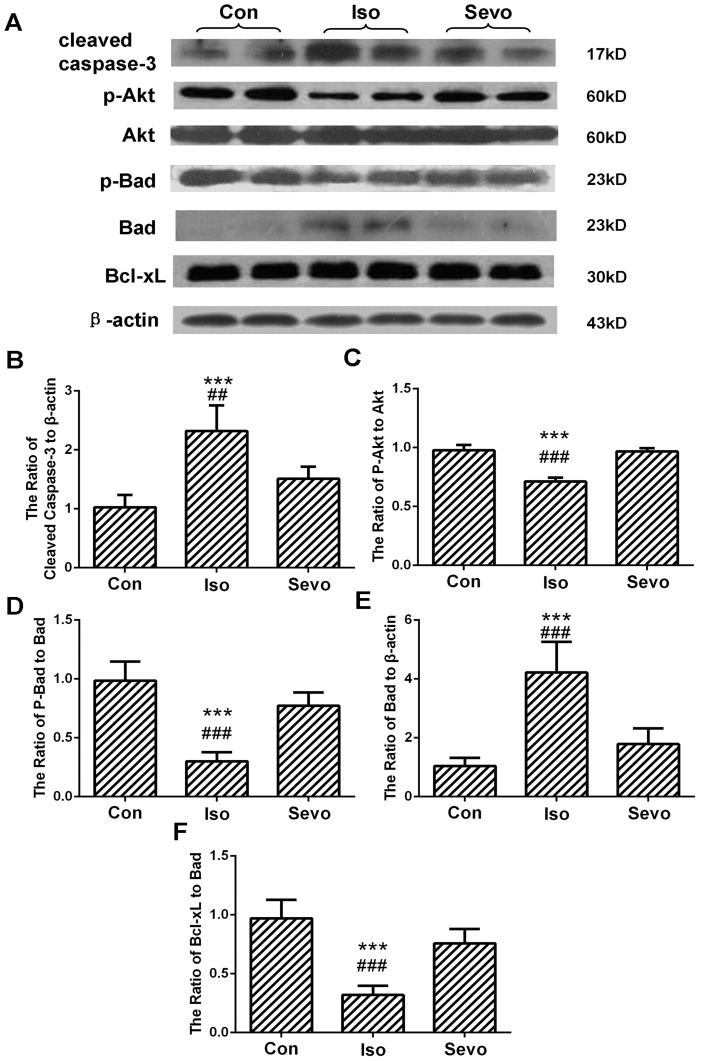
Isoflurane, not sevoflurane, inhibited Akt/Bad signal pathway and decreased Bcl-xL/Bad ratio in the hippocampus of P7 rats (n = 5 in each group). (A) Representative Western blot of caspase-3, phospho-Akt, Akt, phosho-Bad, Bad, and Bcl-xL; (B, C, D, E, F) The quantitative analysis of the cleaved caspase-3 (B), ratio of phospho-Akt to Akt (C), ratio of phospho-Bad to Bad (D), ratio of Bad to β-actin (E) and ratio of Bcl-xL to Bad (F) by one-way ANOVA and post-hoc Bonferronni's correction. Results are the means ± SD. Con: control, Iso: isoflurane, Sevo: sevoflurane. ***P<0.0001 versus control group; ^##^ *P*<0.01, ^###^ *P*<0.001 versus sevoflurane group.

Anesthesia with 0.75% isoflurane induced a significant increase of cleaved caspase-3 positive cells per mm^2^ compared with control animals in rat hippocampal CA1 area (*P*<0.0001, Figs. 1A and B), CA3 area (*P* = 0.0017, Fig. 1C) and DG area (*P* = 0.0022, Figs. 1D) detected by immunohistochemical staining. However, sevoflurane did not induce a significant increase of cleaved caspase-3 positive cells compared with control animals (*P* = 0.071, *P* = 0.105, *P* = 0.152 in CA1, CA3 and DG areas respectively). Quantification of the expression of cleaved caspase-3 protein in whole hippocampus by western blots also revealed that isoflurane significantly induced caspase-3 activation (*P*<0.0001) (Figs. 2A and B), while sevoflurane did not significantly induce caspase-3 activation (*P* = 0.078) (Fig. 2B).

Isoflurane significantly decreased the proteins expression of phospho-Akt (*P*<0.0001, Fig. 2C) and phospho-Bad (*P*<0.0001, Fig. 2D), and increased the expression of Bad protein (*P*<0.0001, Fig. 2E) when compared with control animals. The Bcl-xL expression was similar among the three groups (F = 1.131, *P* = 0.354). The ratio of Bcl-xL/Bad was significantly reduced in isoflurane-treated rats compared with controls (*P*<0.0001, Fig. 2F). There were no significant differences between sevoflurane-treated rats and control rats in the proteins expression of phospho-Akt (*P* = 0.98), phospho-Bad (*P* = 0.051) and Bad (*P* = 0.348) as well as the ratio of Bcl-xL/Bad (*P* = 0.054).

### Dexmedetomidine Reduced Isoflurane-Induced Apoptotic Neurodegeneration

**Figure 3:**
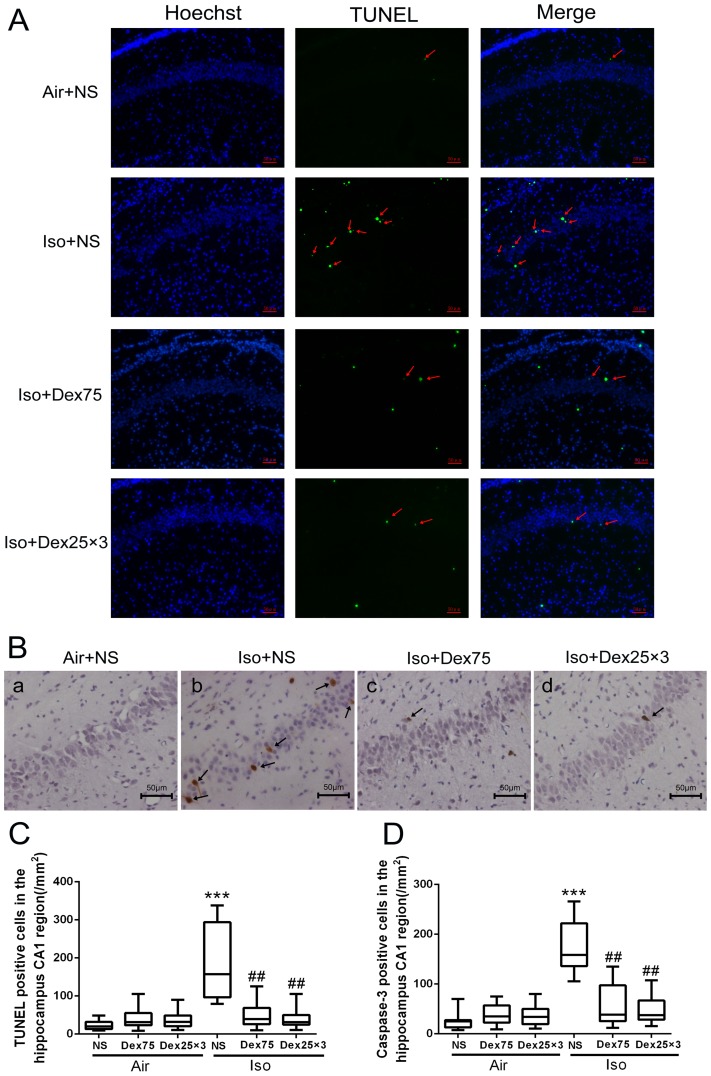
Dexmedetomidine (Dex) inhibited the increase of isoflurane-induced neuroapoptosis in the hippocampus of P7 rats (n = 12 in each group). (A) Representative images of TUNEL in the hippocampal CA_1_ region. Green staining indicated TUNEL-positive cells, blue staining indicated nuclei. Scan bar = 50 µm. (B) Representative images of cleaved caspase-3 immunohistochemical (IHC) staining in the hippocampal CA1 region. Arrows indicate caspase-3 positive cells. Scan bar = 50 µm. (C, D) Quantification of TUNEL positive cells (C) and cleaved caspase-3 positive cells in the hippocampal CA1 region by the Kruskal–Wallis test with the Dunn test for post hoc comparisons. Horizontal lines: group medians; boxes: 25th–75th percentile; whiskers: 5th–95th percentile. NS: normal saline, Dex75: Dex 75 µg/kg, Dex25×3: Dex at 25 µg/kg for three times, Iso: isoflurane. ***P<0.0001 *versus* Air+NS; ^##^ *P*<0.01 *versus* Iso+NS.

**Figure 4:**
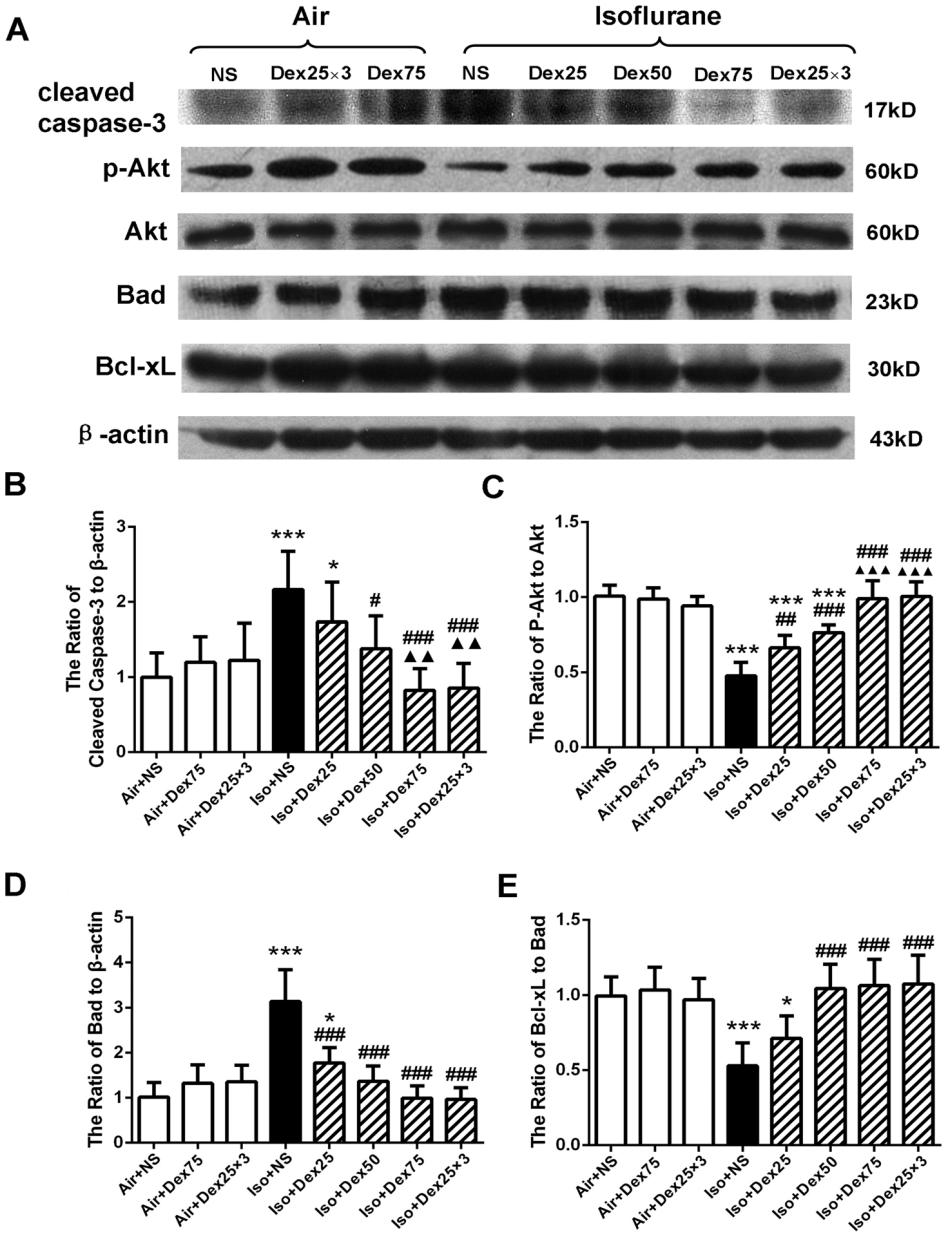
Dexmedetomidine (Dex) reversed isoflurane-induced inhibition of Akt activity and decrease of Bcl-xL/Bad ratio in the hippocampus of P7 rats (n = 7 in each group). (A) Representative Western blot of caspase-3, phospho-Akt, Akt, Bad and Bcl-xL; (B, C, D, E) The quantitative analysis of cleaved caspase-3 (B), phospho-Akt (C), Bad (D), and the ratio of Bcl-xL to Bad (E) by one-way ANOVA and post-hoc Bonferronni's correction. Results are the means ± SD. NS: normal saline, Dex75: Dex75 µg/kg, Dex25×3: Dex at 25 µg/kg for three times, Dex25: Dex25 µg/kg, Dex50: Dex50 µg/kg, Iso: isoflurane. *P<0.05, ***P<0.0001 *versus* Air+NS; ^#^ *P*<0.05, ^##^ *P*<0.01, ^###^ *P*<0.0001 *versus* Iso+NS,; ^▴▴^ *P*<0.01, ^▴▴▴^ *P*<0.001 *versus* Iso+Dex25.

The number of TUNEL positive cells per mm^2^ in the hippocampal CA1 region was higher in the isoflurane-treated animals compared with control animals (*P*<0.0001, Figs. 3A and C). Either pretreatment with 75 µg/kg dexmedetomidine or administration of three doses of 25 µg/kg dexmedetomidine during the isoflurane exposure significantly reduced isoflurane-induced increase of TUNEL positive cells in CA1 region (*P* = 0.0084, *P* = 0.0025, respectively, Figs. 3A and C). Application of one dose of 75 µg/kg dexmedetomidine or three doses of 25 µg/kg dexmedetomidine alone did not increase neuronal apoptosis compared with control (*P* = 0.97, *P* = 0.99, respectively). Similarly, after isoflurane exposure, the number of cleaved caspase-3 positive cells per mm^2^ in CA1 region detected by immunohistochemistry staining was also decreased in dexmedetomidine-pretreated animals and in animals received three doses of 25 µg/kg dexmedetomidine compare with animals exposed to isoflurane only (*P* = 0.0073, *P* = 0.0073, respectively, Figs. 3B and D). Pretreatment with a single dose of 50 µg/kg or 75 µg/kg but not the single dose of 25 µg/kg dexmedetomidine or administration of three doses of 25 µg/kg dexmedetomidine during isoflurane exposure significantly reduced isoflurane-induced cleaved caspase-3 activation in whole hippocampus as detected by Western blotting (*P* = 0.026, <0.0001, 0.97 and *P*<0.0001, respectively, Figs. 4A and B). Furthermore, pretreatment with 75 µg/kg dexmedetomidine or administration of three doses of 25 µg/kg dexmedetomidine provided similar and the most potent protection that abolished isoflurane-induced injury. Dexmedetomidine alone did not induce cleaved caspase-3 activation (*P* = 1.0).

### PI3K/Akt Pathway May Be Involved in the Neuroprotection of Dexmedetomidine

Consistent with previous data, isoflurane significantly decreased the protein expression of phospho-Akt (*P*<0.0001); while pretreatment with dexmedetomidine (25, 50 or 75 µg/kg) or administration of three doses of 25 µg/kg dexmedetomidine dose-dependently increased the expression of phospho-Akt compared with the isoflurane-treated rats (*P*<0.0001 for all post hoc comparisons) (Figs. 4A and C). Dexmedetomidine also dose-dependently reduced isoflurane-induced increase of Bad proteins (*P*<0.0001 for all post hoc comparisons) (Figs. 4A and D) as well as recovered the ratio of Bcl-xl/Bad (*P*<0.0001 for all post hoc comparisons) (Figs. 4A and E). Dexmedetomidine pretreatment at 75 µg/kg provided a similar protective effect on preventing isoflurane-induced changes of the expression of above proteins compared with three doses of 25 µg/kg dexmedetomidine. Dexmedetomidine alone did not cause a significant change of phosho-Akt, Bad and the ratio of Bcl-xL/Bad compared with controls.

**Figure 5:**
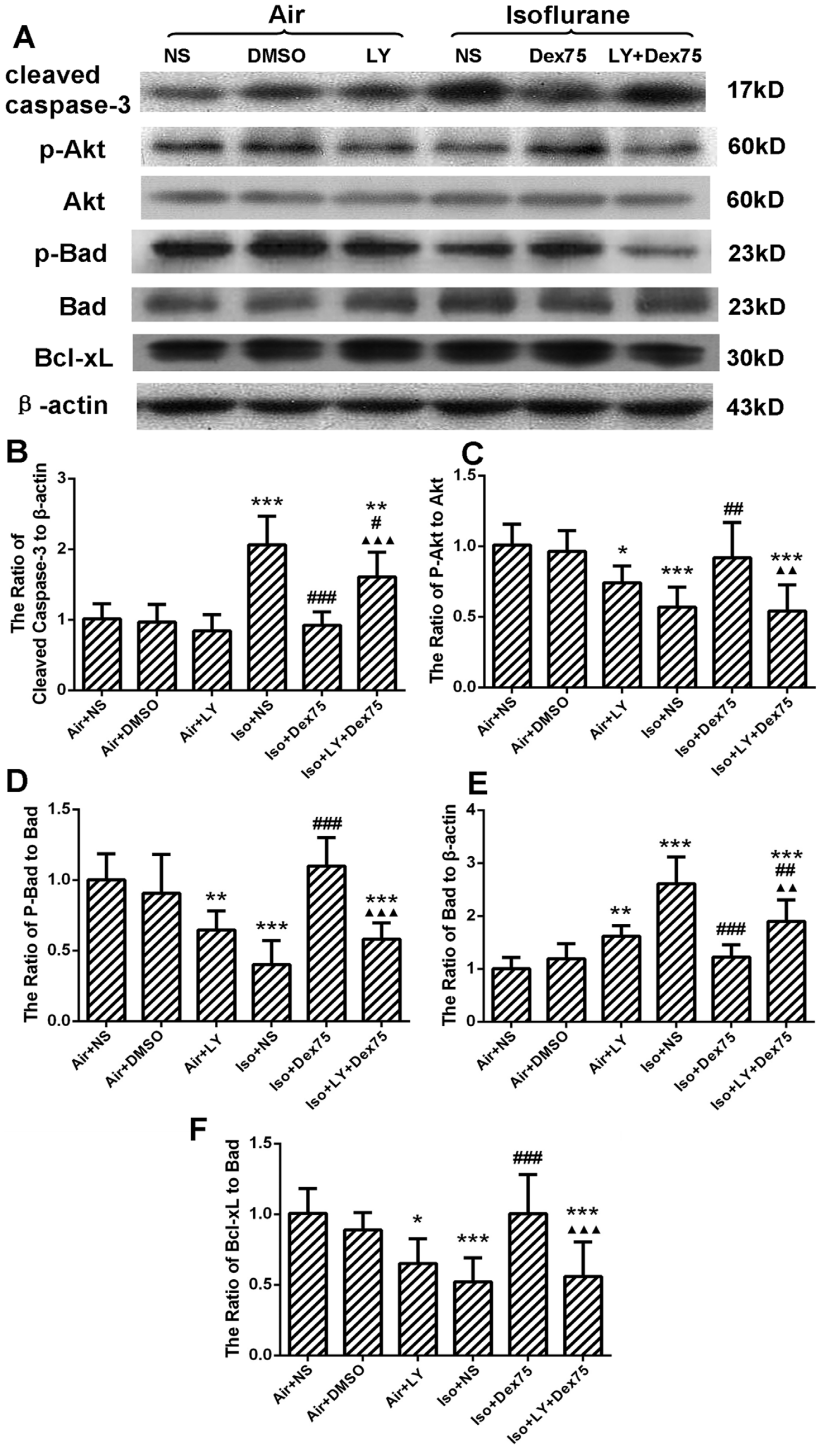
LY294002 partly inhibited protective effect of dexmedetomidine (Dex) pretreatment in the hippocampus of P7 rats (n = 8 in each group). (A) Representative Western blot of cleaved caspase-3, phospho-Akt, Akt, phosho-Bad, Bad and Bcl-xL; (B, C, D, E, F) The quantitative analysis of cleaved caspase-3 (B), ratio of phospho-Akt to Akt (C), ratio of phospho-Bad to Bad (D), ratio of Bad to β-actin (E) and ratio of Bcl-xL to Bad (F) by one-way ANOVA and post-hoc Bonferronni's correction. Results are the means ± SD. NS: normal saline, DMSO: dimethyl sulfoxide, LY: LY294002, Dex75: Dex 75 µg/kg, Iso: isoflurane. *P<0.05, **P<0.01, ***P<0.001 *versus* Air+NS; ^#^ *P*<0.05, ^##^ *P*<0.01, ^###^ *P*<0.001 *versus* Iso+NS; ^▴▴^ *P*<0.01, ^▴▴▴^ *P*<0.001 *versus* Iso+Dex75.

LY294002, a PI3K inhibitor, reversed dexmedetomidine pretreatment-induced neuroprotecion by significantly increasing cleaved caspase-3 expression (*P* = 0.0003, Figs. 5A and B), reducing phospho-Akt (*P* = 0.0011, Figs. 5A and C) and phospho-Bad protein expression (*P*<0.0001, Figs. 5A and D), as well as increasing Bad expression (*P* = 0.0029, Figs. 5A and E). Thus, the ratio of Bcl-xL/Bad was decreased (*P* = 0.0009, Figs. 5A and F). LY294002 alone also inhibited the protein expression of phosho-Akt (*P* = 0.045) and phospho-Bad (*P* = 0.0076), increased the total Bad (*P* = 0.0093) and decreased the ratio of Bcl-xL/Bad (*P* = 0.015), but did not influence the expression of cleaved caspase-3 (*P* = 0.92), when compared with controls.

## Discussion

The present study demonstrated that isoflurane, not sevoflurane, at equivalent low dose, induced neuroapoptosis in the neonatal rat hippocampus and that dexmedetomidine pretreatment provided neuroprotection against isoflurane-induced neuroapoptosis in a dose-dependent manner. Further, isoflurane inhibited the phosphorylation of Akt and Bad, increased the expression of Bad, and downregulated Bcl-xL/Bad ratio; whereas dexmedetomidine reversed those effects. These results indicate that regulating PI3K/Akt signaling may be one of the mechanisms for both isoflurane neurotoxity and dexmedetomidine neuroprotection.

Although anesthetic-induced neurodegeneration has been found in many brain regions, our study focused on hippocampus because previous reports have demonstrated that isoflurane-treated neonatal rats show normal short-term memory, a function predominantly involving the prefrontal cortex, but have an abnormal response to contextual fear conditioning, indicating a severe hippocampal lesion [16]. Besides, Sanders et al. have found dexmedetomidine reduces isoflurane-induced neuroapoptosis to baseline in the hippocampus and thalamus, but not in the cortex, and also prevents hippocampus-dependent neurocognitive impairment after isoflurane exposure [16]. Here, we used hippocampus to further study the mechanism of neuroprotection of dexmedetomidine.

Cleaved caspase-3 expression was used as our marker of apoptosis and cell death because it has been previously validated in this model of anesthetic injury [2], [3], [4], [5], [6], [7]. Both immunohistochemistry and Western blot studies showed that isoflurane, not sevoflurane, at the equivalent dose, increased the expression of cleaved caspase-3 in the hippocampus. Neuronal apoptosis in CA1 was severer than that in CA3 and DG regions detected by IHC. These results were consistent with our previous study in cells [28], [32] and a study in P7 mice [9], indicating that isoflurane has significantly greater potency to cause cell damage compared with sevoflurane at equivalent low doses. However, in another study, there was no difference in cortical apoptosis caused by 2.7% isoflurane and 5.4% sevoflurane for 6 h in neonatal mice [6]. It is possible that there may be methodology-, dose-, species-, and/or brain region-related differences in the apoptotic response.

The mechanisms of inhalational anesthetic-induced neurodegeneration in the developing brain are being extensively investigated. Isoflurane has been demonstrated to be both neuroprotective and neurotoxin via activation of inositol-1,4,5-trisphosphate (IP3) receptors and change of intracellular calcium homeostasis [33], [34], [35]. Limited exposure of isoflurane provides neuroprotection via moderate activation of IP3 receptors and activation of Akt mediated neuroprotection [33]. While prolonged exposure of isoflurane induces neurotoxicity via over activation of IP3 receptors and induction of the excessive Ca^2+^ release from the endoplasmic reticulum [32], [33], [34], [35]. Isoflurane has greater potency than sevoflurane or desflurane to cause calcium release from the endoplasmic reticulum [32], [34]. [Ca^2+^]i overload activates the intrinsic mitochondria-dependent apoptotic pathway, which appears to be an early sign of neuronal injury [36]. Moreover, general anesthesia causes excessive free oxygen radical production, which directly promotes morphological and functional impairment of mitochondria [37]. The regulation of mitochondrial membrane integrity and the release of apoptogenic factors from mitochondria are tightly controlled by the proteins of Bcl-2 family [18]. In this study, isoflurane not only decreased the protein expression of phospho-Akt and phospho-Bad, but also increased the expression of total Bad protein, indicating that isoflurane activates function of Bad via both inhibiting Akt activity and increasing gene transcription of Bad. Isoflurane did not change the expression of Bcl-xL, indicating that isoflurane decreases Bcl-xL/Bad ratio mainly by up-regulating Bad protein. Sevoflurane had no significant effects on the expression of these proteins, which may partly explain why it did not induce neuroapoptosis in rat hippocampus. Our results on the change of Bcl-xL are not consistent with a previous study [36] showing that the expression of Bcl-xl was decreased by a cocktail of anesthetics (0.75% isoflurane, 75 vol% nitrious oxygen and 9 mg/kg midazolam). This cocktail of anesthetics also induced more severe neuroapoptosis than 0.75% isoflurane alone. Our recent study using 1.1% isoflurane also decreased the expression of Bcl-xL [25]. These findings indicate that increased depth of anesthesia further disturbs the expression of Bcl-xL.

Dexmedetomidine is commonly used by a continuous infusion method in pediatric patients for anesthesia and sedation, but sometimes it is also given as a bolus. Besides its hypnotic and analgesic effects, it is found to have neuroprotective effect in several brain injury models [38], [39], [40]. The study by Sanders et al [16] has identified that three doses of 25 µg/kg dexmedetomidine have the maximal neuroprotective effect compared with three doses of 1 µg/kg or 10 µg/kg, and could abolish the injury induced by 0.75% isoflurane for 6 h (the same model as ours) in the hippocampus; whereas single dose of 75 µg/kg dexmedetomidine did not increase neuronal apoptosis. Whether pretreatment of dexmedetomidine will still have neuroprotective effects on long-time isoflurane exposure is underdetermined. Base on Sanders's study, three doses (25 µg/kg, 50 µg/kg and 75 µg/kg) of dexmedetomidine were chosen and administered by intraperitoneal injection.

Consistent with previous study [16], our results showed that three doses of 25 µg/kg dexmedetomidine significantly reversed isoflurane-induced neuronal apoptosis in the hippocampus of neonatal rats. Importantly, pretreatment with dexmedetomidine before isoflurane exposure could also provide neuroprotection in a dose-dependent way. Dexmedetomidine at 75 µg/kg administered either by a pretreatment method or 25 µg/kg for three times provided similar protection that was significantly better than that provided by pretreatment with 25 µg/kg or 50 µg/kg dexmedetomidine. Blood gas and glucose levels in these rats were not monitored because previous studies [17] using the organotypic hippocampal slice culture model also demonstrated the protective effects of dexmedetomidine. These in vitro experiments can control the potential confounding effects of hypoxia, hypercarbia, and abnormal glucose levels and body temperatures that could occur in the in vivo experiments, suggesting that dexmedetomidine can prevent the isoflurane injury by direct action within the central nervous system rather than through physiological alteration mechanisms. The addition of dexmedetomidine probably increased the depth of anesthesia. Our results showed that dexmedetomidine decreased but not increased the neuroapoptosis, which further suggests that dexmedetomidine-induced neuroprotection is not mediated by altering physiological parameters.

It is believed that dexmedetomidine provides its neuroprotection via α2-adrenoceptors, especially the α2A-adrenergic receptor subtype [16], [41]. Since the α2-adrenoceptor antagonist atipamezole only partly reversed neuroprotective effects of dexmedetomidine on isoflurane-induced neurotoxicity in rats [16], other mechanisms may be involved. Our results demonstrated that dexmedetomidine reversed isoflurane-induced inhibition of Akt activity and Bcl-xL/Bad ratio, which may contribute to the increased stabilization of the inner mitochondrial membrane and inhibiting neuroapoptosis provoked by isoflurane. Reversal of dexmedetomidine-induced neuroprotection by the PI3K inhibitor LY294002 in the hippocampus indicates that this neuroprotection is mediated by activating PI3K/Akt pathway. Our results are consist with the study of Zhu et al [42], which has showed PI3K/Akt pathway also participate in the neuroprotection by dexmedetomidine against cerebral ischemia/reperfusion injury in rats. Whether PI3K/Akt signaling activation is via α2-adrenoceptor needs further study. It should also be noted that LY294002 only partly inhibited the neuroprotective effects of dexmedetomidine against isoflurane. Other protective signal pathways, such as activation ERK1/2 via imidazoline receptors [13], may also be mechanisms for the dexmedetomidine's neuroprotective effect.

There are limitations in this study. Firstly, we did not measure the plasma levels of dexmedetomidine. However, intraperitoneal injection usually results in good plasma levels. Also, we only studied short-time effects in the hippocampi of neonatal rats. Additional experiments are needed to study the long-term effects and to test the impact of dexmedetomidine pretreatment on isoflurane-induced injury in other brain regions and cognitive dysfunction. Finally, whether dexmedetomidine is effective to reduce sevoflurane-induced neurotoxicity also needs further study.

In conclusion, isoflurane, not sevoflurane, induced neurotoxity by inhibiting Akt activity. Dexmedetomidine pretreatment provided neuroprotection against isoflurane-induced neuroapoptosis in a dose-dependent manner by preserving PI3K/Akt pathway. There is still unresolved clinical relevance of anesthesia-induced neurotoxicity in animal studies to pediatric anesthesia practice and the relative immaturity of the P7 rats compared with term human neonates [43]. Nevertheless, since dexmedetomidine is commonly used clinically for analgesia, sedation and anesthetic sparing, this effect of dexmedetomidine may have great translational potential if a clinical injury from anesthetics is proven in children.

## Supporting Information

Figure S1 **Verification the accuracy of intraventricular injection by methylene blue.** A. Coronal section at 1.0 mm caudal of intraventricular injection. B. Coronal section at 5.0 mm caudal of intraventricular injection. Methylene blue was distributed in both lateral ventricles two minutes after intraventricular injection.
(TIF)
