# Supplementary figures and images for: Dexmedetomidine Reduces Isoflurane-Induced Neuroapoptosis Partly by Preserving PI3K/Akt Pathway in the Hippocampus of Neonatal Rats
Source: PLoS One. 2014 Apr 17;9(4):e93639. doi: 10.1371/journal.pone.0093639 (PMC3990549; doi:10.1371/journal.pone.0093639)

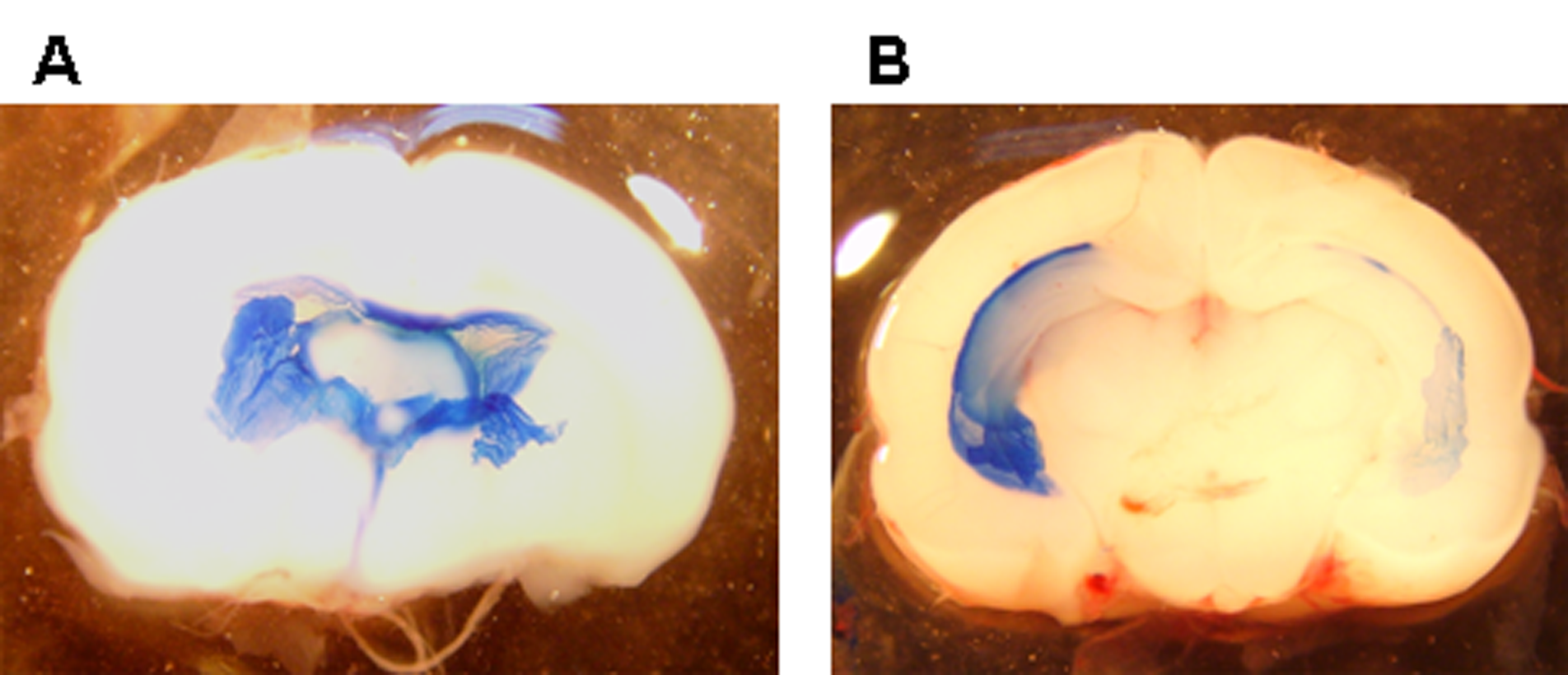

Supplement: Figure S1 — Verification the accuracy of intraventricular injection by methylene blue. A. Coronal section at 1.0 mm caudal of intraventricular injection. B. Coronal section at 5.0 mm caudal of intraventricular injection. Methylene blue was distributed in both lateral ventricles two minutes after intraventricular injection. (TIF) [file pone.0093639.s001.tif]
